# Assessment of right ventricular function in patients after coronary artery bypass graft: a single center study

**DOI:** 10.1038/s41598-025-25795-7

**Published:** 2025-11-23

**Authors:** Salma Taha, Ahmed Taha, Maria Refaat, Hussein El Khayat, Salah Atta, Ahmed Mandour, Ahmed Abdel-Galeel

**Affiliations:** 1 Department of Cardiovascular Medicine, Assiut University Heart Hospital, Assiut University, Assiut, 71526 Egypt; 2 Department of Cardiothoracic Surgery, Assiut University Heart Hospital, Assiut University, Assiut, Egypt; 3 Department of Anesthesia and ICU, Assiut University Hospitals, Assiut University, Assiut, Egypt

**Keywords:** RVD, CABG, STE, Cardiology, Diseases, Medical research

## Abstract

Right ventricular dysfunction is a major risk factor in coronary artery disease. In patients undergoing revascularization for left ventricular ischemia, the incidence of RVD is reported in about 20%. This study aimed to assess right ventricular function as a possible risk factor in patients undergoing coronary artery bypass graft. This prospective study was conducted on 77 patients who underwent coronary artery bypass grafting. All cases were subjected to detailed medical history, full physical examination, electrocardiogram, routine laboratory tests including echocardiography or STE, and all parameters obtained before, within one week, and six months after surgery. Though LVEF preoperatively was comparable between the two study groups, LVEF one-week postoperatively was significantly lower in the RVD group compared to non RVD group; at the same time, there was a significant reduction in LVEF in both groups one week postoperatively compared to preoperatively, and this was more obvious in RVD group compared to non RVD group. Moreover, RVD developed shortly after CABG in less than one-third of our study population, but it recovered in most of them soon at the 6-month follow-up visit. It is suggested that in the early days after the CABG surgery, there is a decline in right ventricular function, which is relatively reversible at longer intervals (6 months after surgery). The right ventricular global longitudinal strain is a reliable way to address RVD.

## Introduction

Right ventricular dysfunction (RVD) is an important risk factor for coronary artery disease (CAD).) patients, and approximately 20% of CAD patients undergoing revascularization for coronary artery ischemia have this combination^1^. RVD is a potential factor contributing to heart failure following cardiac surgery, and it is associated with a significant mortality rate^2,3^. RVD is a recognized cause of hypotension early after coronary artery bypass graft surgery (CABG)^3–5^.

A decrease in RV function is an event known to occur after CABG. RVD can be seen during and immediately after cardiac surgery. Although the mechanism of this phenomenon is not well understood. Cardiopulmonary bypass (CPB), perioperative myocardial ischemia, myocardial damage during operation, prolonged cardioplegia, and pericardial disruption or adhesion have been suggested as probable causes^4–6^.

Major reasons for complications of cardiac surgery are the need for hypothermic cardiac arrest, aortic cross-clamping, and exposure to a CPB^7,8^. It has been postulated that avoidance of these factors by performing off-pump coronary artery bypass (OPCAB) surgery might reduce perioperative morbidity and improve outcomes^7^. The portion of CABG on the beating heart without the use of CPB has been expanded in cardiac surgery as a result of awareness of the damaging effect of CPB^8,9^.

A few studies focused on hemodynamic abnormalities associated with OPCAB indicated that the main mechanism of hemodynamic derangements is the diminished function of both ventricles during coronary artery anastomosis. And, especially, impaired function of the RV^10–12^. It is reported that the major cause of hemodynamic changes during OPCAB was disturbed diastolic filling of the RV through the measurement of chamber pressures or monitoring of echocardiography^11^. However, clinical studies evaluating the change in RV function in patients with ischemic heart disease after CABG surgery are very rare.

This study aimed to evaluate the effect of CABG, either on-pump coronary artery bypass (ONCAB) or OPCAB, on the RV function using conventional echocardiography and speckle tracking echocardiography (STE). To our knowledge, it is the first study to assess the effect of CABG surgery on RV function using STE with relatively long follow-up periods.

## Patients and methods

This prospective study was conducted at Assiut University Heart Hospital from May 2019 to April 2021. Sample size calculation was conducted using G*Power 3 software. A calculated minimum sample of 60 patients was needed to detect an effect size of 0.25 in the variance of RVD on three points of time (baseline, 1-week, and 6-months) repeated measurements with 0.5 correlation among repeated measurements and no sphericity correction Ɛ, with an error probability of 0.05 and 80% power.

Patients who underwent elective CABG of different age groups and of both sexes were included, while those with abnormal cardiac rhythm, poor echo window, prior RV dysfunction, and left ventricular (LV) dysfunction (ejection fraction (EF) < 40%) or refused to participate were excluded. All patients were subjected to the following:

- **Clinical evaluation**, including thorough history taking focusing on socio-demographics such as age and sex. Other relevant clinical data such as smoking, diabetes mellitus (DM), hypertension (HTN), previous myocardial infarction (MI), previous percutaneous coronary intervention (PCI) procedures, and history of respiratory troubles were reported. A full physical examination was conducted.
- ** 12 leads ECG** to ensure sinus rhythm.
- **Echocardiography** includes conventional and STE. An echocardiographic assessment was carried out before surgery to confirm fulfilling the inclusion criteria of the study, a one-week follow-up assessment, and a final 6-month follow-up visit.

### *Conventional echocardiography*

All enrolled patients had transthoracic echocardiography according to the American Society of Echocardiography (ASE) guidelines^13^, which was done preoperative, early post-operative (within one week), and six months post-operative by the same operator and the same machine (Phillips healthcare Epic 7 C, release 1.7.1) using S5-1 probe for 2D data. Tricuspid annular plane systolic excursion (TAPSE) was measured using M mode at lateral tricuspid annulus in apical four-chamber view. A modified apical four-chamber view focused on RV was used to measure RV area by tracing the RV endocardium during systole and diastole to calculate right ventricular fractional area change (RVFAC). Pulsed wave Doppler was used to measure right ventricular systolic velocity (RVS´) in the subcostal four-chamber view by placing tissue Doppler image (TDI) sample volume at the lateral tricuspid annulus. Calculated volumes measured RV stroke volume, end-systolic volume (ESV), end-diastolic volume (EDV), and heart rate (HR).

### *Speckle tracking echocardiography*

Apical four-chamber views were specifically optimized to visualize the RV and obtain echocardiographic cine loops by recording three consecutive heart cycles (> 61 frames per second). Data was stored in DICOM format, and offline analyses were performed using QLAB 10.4 software (Philips Healthcare). After entering the aCMQ interface to determine the apical four-chamber view and choosing the AP4 option, a region of interest (ROI) was traced by tracing the endocardial border on an end-diastolic frame by clicking three separate points (apex, lateral, and septal points of the tricuspid annulus) at end-diastole in the RV from the RV-focused view. The ROI was automatically estimated and adjusted to fit the thickness of the RV-free wall and the septum. Adequate tracking was verified in real time. To ensure optimal tracking, the ROI was adjusted, or the contour was manually corrected. Special care was taken to fine-tune the ROI using visual assessment during cine loop playback to ensure that the segments were tracked appropriately.

* According to the latest recommendations, right ventricular global longitudinal strain (RVGLS) above − 14% is likely to be abnormal, so we considered the value of − 14% as a cut-off point for RVD diagnosis^14^.

- **CABG Surgery**: All study patients underwent CABG surgery, either ONCAB or OPCAB, according to patients’ clinical data and surgeon preference. All patients received left internal mammary artery (LIMA) anastomosis to the left anterior descending coronary artery (LAD). According to the site of coronary artery lesions, some patients received saphenous venous graft (SVG) to obtuse the marginal artery (OM) and/or right coronary artery (RCA).

### Statistical analysis

Data were verified, coded by the researcher, and analyzed using IBM-SPSS 24.0 (IBM-SPSS Inc., Chicago, IL, USA). Descriptive statistics: means, standard deviations, medians, ranges, frequency, and percentages were calculated. Test of significances: Chi-square/Fisher’s exact/Monte Carlo Exact test was used to compare the difference in the distribution of frequencies among different groups. For normality testing, a Kolmogorov-Smirnov test indicated that the main continuous variables under study followed a normal distribution, D (77) = 0.168 − 0.071, *p* = 0.159–0.201). Student t-test analysis was carried out to compare the means of dichotomous data. A paired sample t-test analysis was carried out to compare the means of dichotomous data using repeated measures. A significant p-value was considered when it was < 0.05.

### Ethical consideration

The study protocol was approved by the Medical Ethical Committee, Faculty of Medicine, Assiut University under IRB number 17100309, on 09.09.2017. Trial registration was prospectively undertaken on clinicaltrial.gov (NCT03275220) on 07.09.2019. The study was carried out in accordance with the Helsinki Declaration guidelines.

A written informed consent was obtained from the patient before participating in the study. All collected data was confidential and was used for scientific research only. Every research participant had the complete right and freedom to withdraw from study at any time without any consequences on the medical service provided.

## Results

**Fig. 1:**
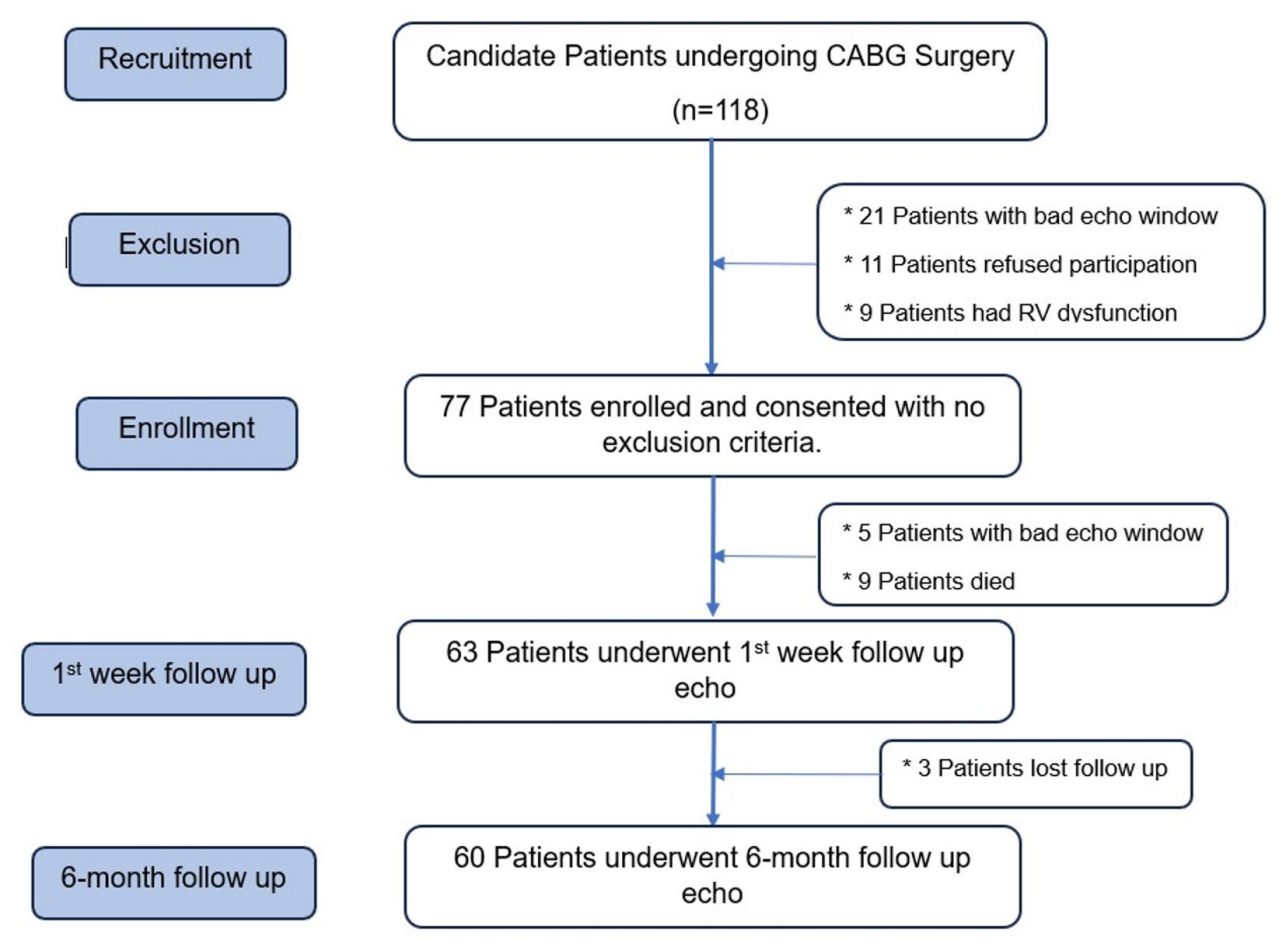
Flow chart of the study population.

One hundred eighteen patients underwent elective CABG surgery at Assiut University Heart Hospital during the study period. Seventy-seven patients were enrolled and consented to participate in the study. Fourteen patients didn’t undergo 1st -week follow-up echo. Finally, only 60 patients succeeded in completing a 6-month follow-up, Fig. 1.

Table 1 summarizes the demographic, clinical, operative, and outcome data of the study population.

**Table 1:** Demographic, clinical, operative, and outcome characteristics of the studied sample.

Variable	*n* = 60
Age in years (Mean ± SD)	55.97 ± 9.42
Sex (Male, %)	48 (80)
DM (%)	32 (53.3)
HTN (%)	29 (48.3)
Smoking (%)	44 (73.3)
Previous MI (%)	9 (15)
Previous PCI (%)	5 (8.3)
Previous respiratory troubles	4 (6.6)
Operative technique	
ONCABG (%)	40 (66.7)
OPCABG (%)	20 (33.3)
Graft type	
LIMA	11 (18.3)
LIMA + SVG to OM	30 (50)
LIMA + SVG to RCA	19 (31.7)
Cross clamping time (Mean ± SD) in min	65 ± 28
Outcome at 1-week follow-up	
RVD	17 (28.3)
Normal	43 (71.7)
Outcome at 6-month follow-up	
RVD	4 (7)
Normal	56 (93)

DM: diabetes mellitus; HTN; hypertension; MI: myocardial infarction; PCI: percutaneous coronary intervention; ONCAB: on−pump coronary artery bypass; OPCAB: off−pump coronary artery bypass; LIMA: left internal mammary artery; SVG: saphenous venous graft; OM: obtuse marginal artery; RCA: right coronary artery; RVD: right ventricular dysfunction.

Though 63 patients underwent the initial follow-up one-week post-operative, we consider only 60 patients who completed the 6-month follow-up visit and we excluded the three patients who didn’t complete the 6-month follow up protocol. At 1-week post-operative follow-up, there were 43 patients with normal RV function and 17 patients with RVD according to RVGLS estimation. Patients with RVD received anti-failure treatment, mainly diuretics, mineralocorticoid inhibitors, and vasodilator therapy. At a 6-month follow-up visit, there were only four patients with RVD; the vast majority of RVD patients diagnosed initially at a one-week follow-up visit recovered after appropriate anti-failure therapy.

According to 1-week follow-up RVGLS results, we divided our study population into two study groups:

(A) Group I: It consists of 43 patients (71.7%) with RVGLS below − 14% and is called the non-RVD group.
(B) Group II: It consists of 17 patients (28.3%) with RVGLS equal to or above − 14% and is called the RVD group.

Table 2 shows the difference between the two groups.

**Table 2:** Relationship between baseline characteristics and RVD at 1-week follow-up.

	Normal (*n* = 43)	RVD (*n* = 17)	*P*-value
Age (mean ± SD)	55.65 ± 10.3	56.76 ± 7.0	0.68*
Sex (male, %)	32 (74.4)	16 (94.1)	0.08**
DM (%)	24 (55.8)	8 (47.1)	0.540**
HTN (%)	22 (51.2)	7 (41.2)	0.485**
Smoking (%)	29 (67.4)	15 (88.2)	0.01**
Previous MI (%)	5 (11.6)	4 (23.5)	0.043***
Previous PCI (%)	2 (4.3)	3 (17.6)	0.065***
Previous respiratory troubles	3 (6.9)	1 (5.9)	0.654**
Operative technique			0.026*
ONCABG	25 (58.1)	15 (88.2)	
OPCABG	18 (41.9)	2 (11.8)	
Graft type			0.309****
LIMA	8 (18.6)	3 (17.6)	
LIMA + SVG to OM	23 (53.5)	7 (41.2)	
LIMA + SVG to RCA	12 (27.9)	7 (41.2)	
Cross clamping time (Mean ± SD)	65 ± 26	66 ± 25	0.456*

Significant values are in [bold].

* Independent sample T−test was used to compare means between groups.

** The Chi−square test was used to compare differences in frequency between groups.

*** Fisher’s Exact test was used to compare differences in frequency between groups.

**** The MCE test was used to compare differences in frequency between groups.

DM: diabetes mellitus; HTN; hypertension; MI: myocardial infarction; PCI: percutaneous coronary intervention; ONCAB: on−pump coronary artery bypass; OPCAB: off−pump coronary artery bypass; LIMA: left internal mammary artery; SVG: saphenous venous graft; OM: obtuse marginal artery; RCA: right coronary artery; RVD: right ventricular dysfunction.

Table 3 shows the echocardiographic variables of our study groups.

**Table 3:** Comparison of echo parameters between the studied groups.

Variable	No-RVD (*n* = 43)	RVD (*n* = 17)	*P*-value*
LVEF % (Mean ± SD)			
Pre-	56.93 ± 6.8	57.35 ± 7.5	= 0.835
1-Week PO	55.02 ± 5.3	51.82 ± 5.1	**= 0.033**
P-value**	< 0.001	< 0.001	
TAPSE (Mean ± SD)			
Pre-	21.77 ± 3.1	19.23 ± 3.0	= 0.067
1-Week PO	17.53 ± 3.3	12.79 ± 2.8	**= 0.010**
P-value**	< 0.001	= 0.011	
FAC % (Mean ± SD)			
Pre-	45.94 ± 9.9	44.24 ± 9.2	= 0.09
1-Week PO	38.26 ± 8.3	32.65 ± 5.1	**= 0.036**
P-value**	< 0.001	< 0.001	
TDIS (Mean ± SD)			
Pre-	11.94 ± 2.4	11.38 ± 2.4	= 0.427
1-Week PO	10.39 ± 1.8	8.47 ± 1.6	**= 0.001**
P-value**	< 0.001	= 0.001	
RVGLS (Mean ± SD)			
Pre-	-23.02 ± 2.1	-22.65 ± 2.1	= 0.12
1-Week PO	-18.47 ± 3.2	-12.94 ± 2.1	**< 0.001**
P-value**	= 0.013	< 0.001	

Significant values are in [bold].

* An independent t−test was used to compare the mean differences.

** A Paired Sample t−test was used to compare the mean differences.

LVEF: left ventricular ejection fraction; TAPSE: tricuspid annular plane systolic excursions; FAC: fractional area change; TDIS: tissue Doppler imaging S wave; RVGLS: Right ventricular global longitudinal strain.

By conventional echocardiography, there was no statistically significant difference between the two groups regarding LVEF, TAPSE, and TDIS. However, at one week postoperatively, they were significantly lower in the RVD group compared to non RVD group (*p* = 0.03, 0.01, and 0.001, respectively); at the same time, there was a significant reduction in these parameters in both groups at 1-week Postoperatively, there was a more pronounced difference in the RVD group compared to the non-RVD group, as compared to the preoperative state, Table 3.

FAC was normal among both groups of the study preoperatively, with no statistically significant difference between them. However, after one week postoperatively, FAC was significantly lower in the RVD group than in the non-LVD group (*p* = 0.036). There was a significant reduction in FAC in both groups one week postoperatively compared to preoperatively, Table 3.

**Fig. 2:**
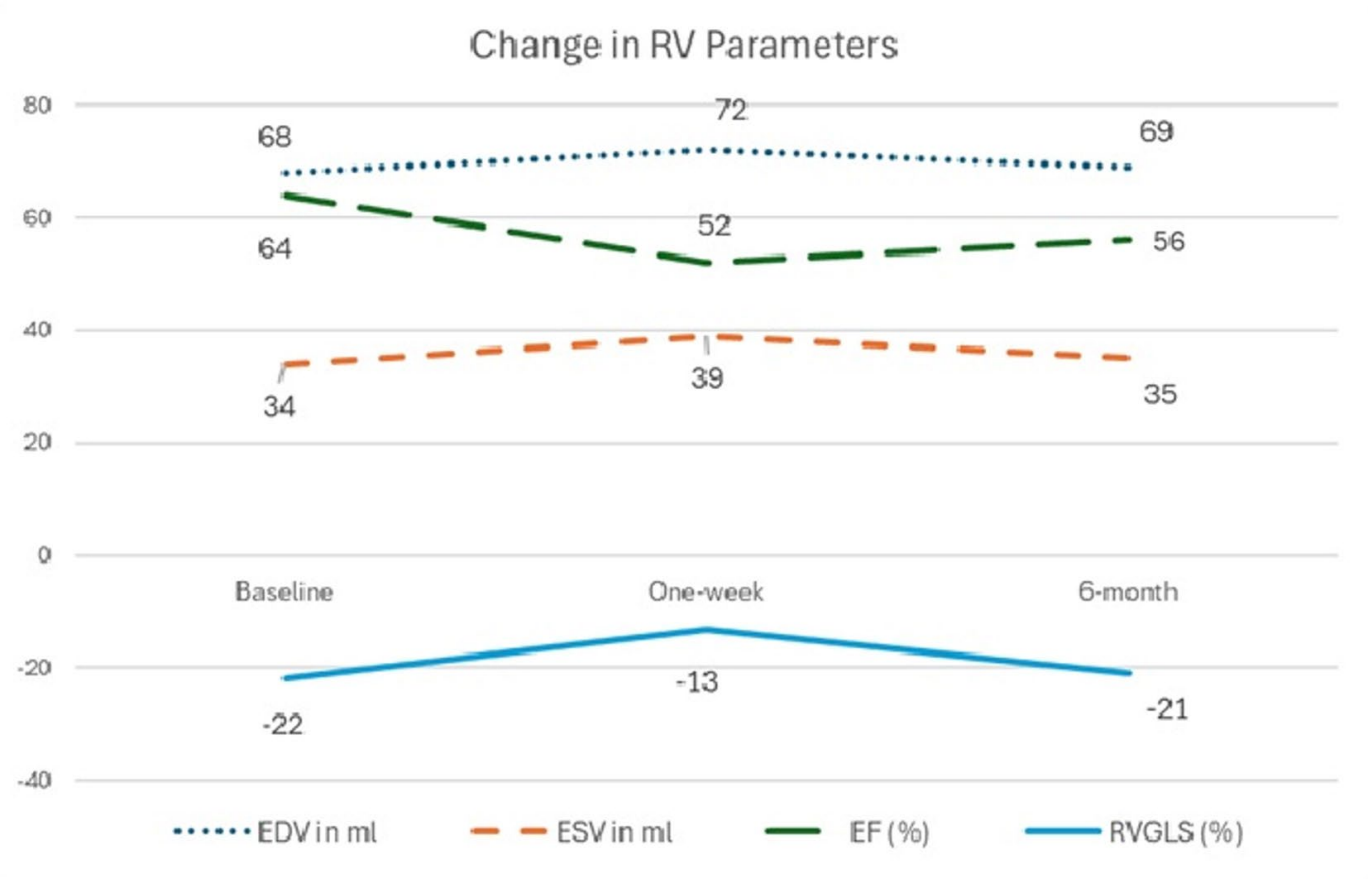
Change in right ventricular hemodynamic parameters after CABG surgery.

Figure 2 shows the change in right ventricular hemodynamic parameters during our study follow-up visits. During a one-week follow-up visit, we noticed a statistically significant increase in mean EDV (68.4 ± 15.1 vs. 78.9 ± 16.3 ml, p-value 0.001) and ESV (34.2 ± 12.2 vs. 44.8 ± 14.8 ml, p-value 0.001). We also detected a statistically significant decline in right ventricular FAC (45.1 ± 8.1 vs. 36.7 ± 9.2 ml, p-value 0.001) and RVEF (59.4 ± 5.4 vs. 49.3 ± 4.3, p-value 0.001) in one-week follow up visit. Using STE, the mean RVGLS was comparable between both groups preoperatively (*p* = 0.12); however, the mean RVGLS was significantly higher in non-RVD patients than RVD patients at 1-week postoperatively (*p* = 0.001), but there was significant reduction in both groups at 1-week postoperatively compared to preoperatively and this was more obvious in RVD group (*p* = 0.001), Table 3.

In an attempt to identify the proper predictors for RVD development early post CABG surgery, we drew a multi-variate logistic regression analysis. Table 4 showed the multivariable logistic regression model for the predictors of RVD at 1st week. After adjusting for all correlates, the final model contained three independent predictors (sex, previous PCI% and operative technique). In other words, it was found that males had 6.5 times (AOR = 6.478, 95% CI; 1.159–13.430, *p* = 0.034) increase in the risk of RVD at 1st week compared with females. Also, cases with history of previous PCI had 4.1 times (AOR = 4.102, 95% CI; 1.824–11.284, *p* = 0.047) more possibility of having RVD at 1st week. Likewise, the chances of having RVD at 1st week were reduced by 46% (AOR = 0.535, 95% CI; 0.123–0.991, *p* = 0.042) in cases underwent OPCABG surgical technique compared with those underwent ONCABG surgical technique.

**Table 4:** Predictors of RVD at 1st week: multivariable logistic Regression.

	AOR (95% CI) *	*P*-value
Age/years	1.044 (0.963–1.131)	= 0.297
Sex (Male)	6.478 (1.159–13.430)	**= 0.034**
DM (%)	0.332 (0.079–1.397)	= 0.133
HTN (%)	1.034 (0.460–4.113)	= 0.762
Smoking (%)	1.285 (0.229–3.201)	= 0.471
Previous MI (%)	3.515 (0.552–9.347)	= 0.183
Previous PCI (%)	4.102 (1.824–11.284)	**= 0.047**
Previous respiratory troubles	0.841 (0.066–2.924)	= 0.767
Operative technique		
ONCABG	1 (Ref.)	
OPCABG	0.535 (0.123–0.991)	**= 0.042**
Graft type		
LIMA	1 (Ref.)	
LIMA + SVG to OM	0.710 (0.144–3.501)	= 0.374
LIMA + SVG to RCA	1.256 (0.245–6.445)	= 0.584
Cross clamping time	1.024 (0.427–2.123)	= 0.664

Significant values are in [bold].

AOR, Adjusted Odds Ratio; CI, Confidence Interval.

## Discussion

Myocardial revascularization in patients with CAD is a unique solution for better symptom relief and outcome improvement. In some instances, CABG surgery remains the sole option to solve a condition with critical multi-vessel CAD with a high SYNTAX score and/or left main stem lesions^15^. However, CABG surgery is a major surgery with considerable perioperative morbidity and mortality hazards, so every effort should be made to identify better the possible risk factors that increase such hazards. There is no doubt that the development of post-operative RVD is associated with significant complications and poor prognosis^16^. Many studies have addressed the issue of RVD development after CABG surgery and associated such conditions with poor outcome^16^. In a retrospective large-scale study involving more than 1000 patients who underwent cardiac surgery, RVD was independently associated with 2-years all-cause mortality^17^. A recent and somewhat small prospective experiment found that there was a notable decrease in RV function and a decline in exercise capacity following CABG. This indicates that cardiologists should give greater consideration to evaluating the RV during follow-up appointments with patients who have undergone CABG^18^.

However, the assessment of RV function remains an obstacle for many cardiologists. Echocardiographic imaging of the RV is a challenge due to its complex anatomic structure^19^. Therefore, additional tools to overcome the inaccuracy of the commonly used techniques are strongly demanded to assess RV function perfectly. STE of the RV is reported to have high accuracy and reproducibility and to be superior to the traditional RV contraction indices^20^. RV strain analysis has demonstrated more sensitivity in detecting mild myocardial dysfunction compared to standard measures, making it a more effective imaging method^21^.

Some authors evaluated some parameters to assess the RV function after CABG surgery, including tricuspid annular motion (TAM), tricuspid annular velocity (TAV), and RV strain. They reported that RV strain should be applied as an alternative to TAM and TAV in the assessment of RV function, particularly when there are limitations in the application of TAM and TAV post-CABG surgery^22^. RV longitudinal strain, as measured by STE, has been demonstrated to be a valuable two-dimensional echocardiographic technique for identifying compromised RV function. This method has shown associations with many clinical and echocardiographic characteristics^19^.

Our study reliably assessed RV global strain as a tool for the determination of RV function after CABG surgery. The main finding of the current study is that about one-third of patients with normal RV function who underwent CABG developed RV dysfunction 1-week postoperatively, and fortunately, 75% of them improved at 6-month follow-up. Similarly, Chinikar et al. reported RV dysfunction one week after CABG surgery in 62–81% at one-week follow-up and 37–49% at a 6-month visit, depending on the method of assessment of RV function. However, none of their assessment tools included tissue Doppler imaging^18^. Similarly, Ordienė et al. studied the changes in biventricular function after CABG surgery in 81 CABG patients and found that the reduction was seen right after the surgery and had a tendency to improve within the follow-up period (6 months)^23^. Also, John et al. reported a significant increase in the RV function post-CABG over the next two months after surgery^24^.

On the other hand, in another prospective study involving more than 200 CABG patients, they reported worsening of RV function at one-year follow-up. This worsening was more evident among those who underwent RCA revascularization^19^. In a study conducted on 160 CABG patients, they demonstrated an increase in the frequency of right ventricular systolic and diastolic dysfunction 18 months after CABG^25^. Likewise, among 42 patients who were eligible for CABG and included in a study by Hashemi et al. on the impact of CABG on RV function, TAPSE reduced substantially after CABG, with a significant decline in RV peak systolic velocity as well^26^. Rong et al. studied RV function after open heart surgery (15% of them had only CABG surgery) by 2D speckle tracking and found that there was a significant reduction in RV function postoperatively^27^.

Similarly, Gozdzik et al. enrolled 69 patients scheduled for CABG; they observed a significant decrease in the RVGLS postoperatively^28^. Also, among 24 patients undergoing elective CABG enrolled by Bitcon et al., there was a significant decrease in RV-free wall strain^29^. In 2010, Yadav et al. published a study with two parts: a retrospective arm including 101 patients (median CABG duration was ten years) and a prospective arm including only 20 post-CABG patients (follow-up was three months post-operative). This study suggested that the effects of CABG surgery on the right ventricle may be permanent. This effect was shown in patients whose surgery was a decade ago and was also readily demonstrable in patients undergoing surgery at that time. However, they didn’t understand the mechanism of such dysfunction^30^. We claim that this study was mostly retrospective in nature; it didn’t provide true full data about the recruited patients, their background history, and their medical data. We reported that in our study, we treated those patients who developed RVD readily after diagnosis using conventional anti-failure treatment, and this resulted in improvement in 75% of them. Their study didn’t show whether those patients received anti-failure treatment or not and what that treatment was. Moreover, in the prospective arm, the follow-up duration was too short to show any possible improvement in RV function.

In our study, the univariate analysis showed that the development of RVD after CABG surgery was associated with smoking, a history of previous MI, and having surgery using the OPCAB technique. However, the multivariate model showed that male gender, history of previous PCI and using ONCAB surgical technique were associated with the development of RVD after surgery.

For smoking as a risk factor, smokers significantly represented the majority (15 patients, 88.7%) of those who developed RVD after surgery. Smoking is associated with worse outcomes after cardiac surgery^31^. Moreover, smoking was highlighted to be one of the most modifiable risk factors for post-operative morbidity and mortality after CABG surgery^32^. Persistent smokers were shown to have a greater incidence of pulmonary problems after CABG than non-smokers, according to Ji et al. It was anticipated that quitting smoking more than a month prior to surgery may lessen early significant morbidities after CABG surgery. The reported pulmonary complications could be attributed to the disturbance in RV function^33^.

History of previous myocardial infarction was demonstrated to have a relationship with the development of RVD after CABG surgery. In many studies, there was an association between the history of prior myocardial infarction and worse outcomes after CABG surgery. In a large cohort study that extended up to 9 years follow-up, the mortality in patients who did not have MI was low. Compared to the non-MI cohort, patients who had an MI prior to undergoing a CABG had significantly higher mortality^34^. As for RVD, Sumin et al. carried out a study to display the predictors of RVD after CABG surgery. They found that history of myocardial infarction before surgery was notably related to an elevated likelihood of detection of RV diastolic dysfunction 18 months after CABG^25^. On the other hand, Bootsma et al., in their study, didn’t find such an association^17^.

OPCAB has become a common practice for CABG surgery in many countries all over the world, especially in Japan, with approximately 65% of CABG procedures currently being performed using OPCAB^35^. This favorable effect of OPCAB could be attributed to short cross-clamping and cardiac bypass time. Using cardiac magnetic imaging, Pegg et al. investigated the impact of off-pump versus on-pump coronary artery bypass grafting on early and late RV function and discovered a decline in RV function early postoperatively. In contrast to our results, they found that RVD development was irrespective of the surgical technique used. Moreover, there was a complete recovery after six months^36^. In our study, 11 patients (18.3%) of the studied group had only LIMA graft to LAD, 30 patients (50%) had both LIMA graft to LAD artery and SVG to OM artery, and 19 (31.7%) had LIMA graft to LAD artery and SVG to RCA. We didn’t find any association between RVD development after surgery and the performed revascularization procedure during surgery. This is the same as what was reported by Pegg et al., who stated that there was no difference in any parameter of RV function between those receiving a graft and those who did not^36^.

In contrast to these results, Kwak et al. noted that there is no significant change in the RV function and cardiac index during anastomosis of the LAD artery and RCA. However, reduced RV function accompanied by an increase in RV afterload and a decrease in the cardiac output (COP) was observed during anastomosis of the OM artery. The movement of the beating heart for proper positioning during the connection of the graft to the OM artery results in a notable disruption of right ventricular function and a reduction in cardiac output^37^.

The precise mechanism of RVD post-CABG surgery is still understood. Relaxation of the RV-free wall could be impaired due to myocardial edema. It is suggested that the right ventricle (RV) is greatly affected due to the overall damage caused by surgery. However, this is not worsened by the use of an aortic cross-clamp or cardiopulmonary bypass^36^.

The possible reason for the deformation of RV geometry and reduction of TAPSE after open heart surgery is still unclear. Many factors may contribute to this, such as a changed contraction pattern of the interventricular septum, mechanical effects of pericardiotomy, or post-operative adhesions of the RV. Many theories were proposed for explanation of this dysfunction, such as pericardial opening, injury to the right atrium during cannulation, incomplete myocardial protection, and adhesions between the RV and nearby mediastinal structures^38,39^. In a recent study discussing the overall cardiac function after surgery and the possible biventricular interaction. They stated that both RV and LV functions are affected after surgery. The RVD is mostly affected by factors related to pericardial incision and loss of pericardial constraint. In contrast, studies on LV function speculated that the functional affection may be related to myocardial dysfunction because of ischemia-reperfusion injury^40^.

Interestingly, Rösner et al. reported that RV function as assessed by RVEF and RVFAC and cardiac output were not affected by CABG surgery. However, post-CABG tricuspid TAPSE was markedly reduced^41^. Moreover, Larrazet et al. found that RVFAC remained unchanged despite a decline in RV free wall velocity after CABG. They stated that there was only a geometric change after CABG surgery with no change in RV function^42^. In a recent study on 60 patients who underwent CABG surgery with a follow-up period 6 weeks after surgery, they stated that the two major parameters for right ventricular functional assessment, global 2D longitudinal strain and free wall longitudinal strain, may be less affected by CABG and thus could be more reliable as standard parameters^43^.

## Limitations

The study encountered some limitations; it was a single-center study, with 80% of the analyzed study population being males which may endanger the generalizability of its results. Moreover, we relied upon 2D-speckle tracking echocardiography for the assessment of RV function. It would be better to have CMR imaging. CMR is ideal for evaluating the RV as it allows a comprehensive assessment of its anatomy and physiology without most of the restrictions that impede other imaging methods. Despite that most of patients with RVD recovered at the 6-month follow up visit, this may not reflect the full picture of recovery or dysfunction. Large scale studies with prolonged follow up period may be warranted in order to better assess RV function after CABG surgery.

## Conclusion

Our study confirmed that after CABG surgery, there would be a decline in RV function as detected by tissue Doppler imaging. Fortunately, this dysfunction is relatively reversible. Smoking, a previous history of coronary intervention, and having surgery on a pump were associated with the development of RV dysfunction.

## Data Availability

All data generated or analysed during this study are included in this published article. Further data related to this article will be shared on request with the corresponding author.

## Abbreviations

RV: Right ventricle
RVD: Right ventricular dysfunction
CAD: Coronary artery disease
CABG: Coronary artery bypass graft surgery
CPB: Cardiopulmonary bypass
CMR: Cardiac Magnetic Resonance
OPCAB: Off-pump coronary artery bypass
ONCAB: On-pump coronary artery bypass
STE: Speckle tracking echocardiography
LV: Left ventricle
EF: Ejection fraction
DM: Diabetes mellitus
HTN: Hypertension
MI: Myocardial infarction
PCI: Percutaneous coronary intervention
ASE: American society of echocardiography
TAPSE: Tricuspid annular plane systolic excursion
RVFAC: Right ventricular fractional area change
RVS´: Right ventricular systolic velocity
TDI: Tissue Doppler image
ESV: End systolic volume
EDV: End diastolic volume
HR: Heart rate
ROI: Region of interest
RVGLS: Right ventricular global longitudinal strain
LIMA: Left internal mammary artery
OM: Obtuse marginal artery
RCA: Right coronary artery
TDIS: Tissue Doppler imaging S wave
TAM: Tricuspid annular motion
TAV: Tricuspid annular velocity
COP: Cardiac output
